# An AI-Generated Integrated Exercise Addiction Screening Scale (EASS-10): A Methodological Demonstration

**DOI:** 10.3390/bs16050817

**Published:** 2026-05-19

**Authors:** Attila Szabo

**Affiliations:** Department of Psychology and Health Management, Faculty of Health and Sport Sciences, Széchenyi István University, H-9026 Gyor, Hungary; szabo.attila@sze.hu

**Keywords:** artificial intelligence, health, questionnaire, physical activity, psychology

## Abstract

Artificial intelligence (AI) is increasingly used in scientific research, including psychiatry. Exercise addiction (EA) research relies on self-report instruments, most of which lack a functional impairment criterion and cannot account for obsessive passion, potentially contributing to inflated prevalence estimates and limited clinical specificity. This study examined whether Claude AI (Sonnet 4.6) could assist in synthesizing a brief, theoretically grounded screening tool for EA by integrating validated constructs from the Passion Scale (PS), the Exercise Dependence Scale (EDS), and the Exercise Addiction Inventory (EAI). Using structured prompting and instrument upload, Claude AI created a 10-item scale by systematically mapping source-scale content onto established EA frameworks, including the components model and DSM-5 criteria. The instrument included nine Likert-type items and one binary gate for functional impairment to enhance clinical specificity. Although no empirical validation data were collected, a Monte Carlo simulation (N = 500) was used solely to verify that the prespecified unidimensional simulation model produced internally coherent response patterns. Because the simulated data were generated from and analyzed under the same unidimensional model, these results constitute a computational plausibility check rather than empirical structural validation. AI-assisted theoretical synthesis may offer a novel methodological approach to instrument development, though this remains a working hypothesis requiring empirical corroboration. The proposed Exercise Addiction Screening Scale (EASS-10) is presented as a proof-of-concept tool that now requires empirical validation in clinical and exercise populations.

## 1. Introduction

The use of artificial intelligence (AI) in developing psychometric tools is a natural extension of its increasing role in psychiatry. In this field, AI has already analyzed complex, multimodal datasets and uncovered hidden patterns that traditional methods might miss (Sun et al., 2025). Sun et al. (2025) emphasize that AI-powered methods can improve diagnostic accuracy by enabling more detailed taxonomies and revealing construct-level patterns that traditional item-selection methods, which rely on researcher intuition or stepwise statistical heuristics, often overlook. The ability of large language models and machine learning algorithms to combine domain expertise with empirical psychometric data makes them particularly well-suited to the iterative, theory-guided process of item development (Sun et al., 2025), for example, when linking addiction frameworks such as Griffiths’ (2005) components model with Vallerand et al.’s (2003) passion theory. In psychiatric assessment more broadly, AI algorithms could be especially effective at integrating diverse data sources, including theoretical frameworks and established measurement scales, by identifying the optimal item combinations that best explain a target construct. However, Sun et al. (2025) caution that AI should serve as a supportive tool alongside expert clinical and scientific judgment, not as a standalone replacement. Therefore, it is important to evaluate AI-generated content theoretically. Moreover, such AI-generated item selections remain theoretical hypotheses that require empirical validation using item response theory (IRT) and confirmatory factor analysis (CFA) with real-world data before claims of optimality can be substantiated.

This capacity directly addresses one of the central methodological challenges in exercise addiction (EA) research. This area suffers from fragmented assessment across multiple instruments (>30; Chhabra et al., 2026; see also Szabo et al., 2015; Downs et al., 2004). The two most widely used tools (Mónok et al., 2012; Szabo et al., 2015) are the Exercise Addiction Inventory (EAI; Terry et al., 2004; EAI-3; Granziol et al., 2023) and the Exercise Dependence Scale-21 (EDS; Hausenblas & Downs, 2002). However, research has shown that passion, generally assessed with the Passion Scale (PS; Marsh et al., 2013), especially obsessive passion (OP), accounts for substantial variance in EA that is not captured by individual EA assessment tools (Kovacsik et al., 2018; Szabo & Kovacsik, 2019).

It is important to acknowledge the conceptual boundary between exercise dependence (as operationalized by the EDS-21) and exercise addiction (as assessed by the EAI). Exercise dependence derives from DSM-IV substance dependence criteria (Hausenblas & Downs, 2002), whereas exercise addiction aligns with behavioral addiction frameworks (Griffiths, 2005). Despite these terminological differences, both constructs share substantial symptom overlap (e.g., tolerance, withdrawal, loss of control), and empirical research demonstrates high convergent validity between the two instruments (Mónok et al., 2012; Szabo et al., 2015). In the present synthesis, items were drawn from shared symptom domains common to both instruments rather than from construct-specific distinctions.

Within Vallerand et al.’s (2003) Dualistic Model of Passion, OP is conceptualized as a rigid, pressure-driven internalization of the activity (in this case, exercise) accompanied by conflict and diminished control. In contrast, harmonious passion (HP) involves flexible, controlled, autonomous engagement. Research suggests that OP may account for up to 50% of the variance in EA, substantially exceeding the explanatory contribution of HP (Kovacsik et al., 2018). Moreover, when passion, particularly OP, is statistically controlled, many observed exercise group differences in EA attenuate or disappear, raising questions about whether EA represents a distinct psychopathological entity or is largely a reflection of obsessive forms of intense commitment (de la Vega et al., 2016; Szabo & Kovacsik, 2019).

Cross-national evidence further supports the robust predictive role of OP, with individuals classified as at risk typically exhibiting especially high levels of obsessive passion (Chhabra et al., 2024; Szabo et al., 2022). Therefore, it is not surprising that researchers have called for controlling for passion in EA research (de la Vega et al., 2022). Hence, the attempt to integrate passion, especially OP, into EA assessment may be justified. Accordingly, the present study had two objectives: (1) to examine whether AI-assisted theoretical synthesis using Claude AI could integrate obsessive-passion-related constructs with symptom content from the EDS and EAI into a single, clinically grounded 10-item screening tool, through theoretical mapping and construct synthesis rather than simple item adaptation; and (2) to evaluate the computational plausibility of the resulting instrument’s factor structure using Monte Carlo simulation. This principle of expert-guided AI use informed the current instrument development, in which AI-generated item creation was aligned with theoretical frameworks and an evidence-based rationale at each stage.

Overall, these points make a case for using AI to develop the Exercise Addiction Screening Scale (EASS-10), leveraging its potential to identify patterns and integrate information to generate a more comprehensive and clinically relevant screening tool than any current scale could offer on its own. Nevertheless, the potential contribution of the EASS-10 should be interpreted within the methodological scope and limitations of the present study.

It is important to emphasize that this study is explicitly positioned as a proof-of-concept methodological demonstration, not as an empirical validation study. No claim is made that the EASS-10 is ready for clinical use. The absence of empirical data is a deliberate design feature of this first-stage investigation, which aims to determine whether AI-assisted theoretical synthesis can produce a structurally coherent and theoretically grounded instrument suitable for subsequent empirical testing. This approach follows established practice in psychometric research, where theoretical instrument development and content specification precede empirical data collection (DeVellis & Thorpe, 2021). The value of this work’s contribution lies in demonstrating the process and its theoretical product, not in providing evidence of validation.

## 2. Method

Item generation and theoretical mapping were performed using Claude Sonnet (Anthropic, version claude-sonnet-4-6; anthropic.com). Claude Sonnet is a large language model (LLM) with advanced natural language understanding, multi-document synthesis, and domain-reasoning capabilities across scientific and clinical literature. It was chosen because it is an advanced AI system trained in extensive collections of scientific texts, enabling high-precision theoretical triangulation across psychometric frameworks. The model was prompted to operate solely within the scope of open-access, peer-reviewed literature on EA, given the availability of hundreds, if not thousands, of relevant records (Szabo, 2024), and to utilize established psychometric and clinical criteria in its reasoning. The exact prompt (as quoted below), accompanied by the uploading of the three instruments (EDS, EAI, and PS), was:

“Based on the current scholastic and/or academic theoretical and clinical knowledge from the open-access scientific/peer-reviewed papers on exercise addiction, use these three scales to develop a 10-item tool with the most optimal evaluation (can be scale, yes-no, answers, etc.) for screening exercise addiction with high certainty. Justify the inclusion of each of the 10 items.”

To ensure transparency and replicability of the AI-assisted procedure, the following procedural details are provided. The initial item generation required a single structured prompt (quoted above) with three instrument files uploaded simultaneously. Claude AI produced a complete 10-item scale with justifications within a single dialogue turn. Next, three additional iterative dialogue turns were conducted: (1) requesting scoring and interpretation guidelines, (2) challenging the proposed cutoff thresholds from a clinical screening perspective, and (3) requesting clarification on the clinical gate rationale. At each stage, the author (a domain expert in EA research with over 25 years of experience) evaluated the AI-generated output against established theoretical frameworks and empirical findings, accepting, modifying, or rejecting specific suggestions based on alignment with current scholarly knowledge. No competing AI outputs were generated; rather, the single-model output was subjected to expert adjudication at each step. The author retained full editorial control over the final selection of items, wording, and theoretical justification.

Regarding technical reproducibility: Claude Sonnet (version 4.6) was accessed via the Anthropic web interface (claude.ai). The platform does not expose user-adjustable parameters such as temperature or top-p sampling; default inference settings were used. LLM outputs are inherently stochastic, meaning that rerunning the same prompt may yield slightly different item formulations. However, because the final instrument was subject to expert adjudication and theoretical verification at every step, the specific AI output serves as a starting point rather than a deterministic endpoint. The prompt was designed with explicit constraints (open-access literature scope, three specific source instruments, 10-item limit, screening purpose), which substantially narrowed the output space. Full reproducibility of AI-assisted research remains an open methodological challenge that the field must address (Bender et al., 2021).

A note on the epistemological status of AI-generated content is warranted. Large language model outputs are probabilistic text generations derived from statistical patterns in training corpora; they do not constitute clinical reasoning or validated scientific evidence. In this study, AI-generated content served exclusively *as a structured starting point for expert-guided instrument development*. All AI output was critically evaluated, revised where necessary, and verified against peer-reviewed literature by the author before inclusion. Throughout Section 3, direct AI output is enclosed in quotation marks and attributed to the AI model (e.g., “the AI-generated output suggested”). At the same time, the author’s own interpretations and theoretical analyses are presented without quotation marks. This convention ensures that readers can distinguish between AI-generated text and author-endorsed scientific positions throughout the manuscript.

## 3. Results

Claude AI generated the following scale: “Exercise Addiction Screening Scale-10 (EASS-10).” The suggested instruction for individuals being screened is: “*Think about your current exercise behavior. Using the scale below, please indicate how well each statement describes you. There are no right or wrong answers.*” The response scale for Items 1–9 is presented in Table 1. Note that Claude AI uses *a frequency scale* that is at odds with the instructions for participants. The proper instruction wording could be framed as: “*Using the scale below, please indicate how often each statement applies to you.*” Alternatively, the scale can also be tested using a 1-6 agreement-disagreement scale.

The choice of a 6-point response format (without a neutral midpoint) was retained from the AI-generated output and is justified on psychometric grounds: even-numbered scales eliminate the tendency toward neutral responding, which can artificially inflate central tendency and reduce variance in clinical screening contexts (Chyung et al., 2017). This forced-choice format is particularly appropriate for EA screening, where endorsement of even mild symptom frequency is clinically informative and a midpoint response would be ambiguous (i.e., it would be unclear whether “neither agree nor disagree” reflects the absence of the symptom or the respondent’s uncertainty).

The choice between a frequency-based response format (Never to Always) and an agreement-based format (Strongly Disagree to Strongly Agree) has implications for the construct validity of the EASS-10. A frequency format was selected for the initial version because most items describe observable behavioral patterns (e.g., exercising despite injury, increasing intensity) for which frequency of occurrence is the most direct and clinically interpretable metric. Agreement scales, by contrast, are better suited for attitudinal or belief-based items. However, Item 9 (“I have the impression that exercise controls me”) and Item 8 are more experiential and subjective and may be better captured with agreement ratings. Future validation studies should empirically compare both formats to determine which yields superior psychometric properties and clinical utility.

The EASS-10, along with symptoms and theoretical addiction criteria, is presented in Table 2. Finally, Table 3 presents the item-level cross-reference for the EASS-10, mapping each item to its theoretical and diagnostic foundations in EDS-21, all versions of the EAI, the Obsessive Passion subscale of the Passion Scale, and the DSM-5/ICD-11 addiction criteria. The table illustrates that each item is anchored in established components of addiction (e.g., tolerance, impaired control, salience, conflict, and continuance despite harm) while explicitly integrating OP as a key explanatory mechanism. It further shows that the final item functions as a clinical gate requiring significant distress or impairment, aligning the tool with diagnostic standards. The purpose of Table 3 is to document theoretical content validity by design and to illustrate that the EASS-10 is a theoretically synthesized, clinically grounded screening instrument rather than a *de novo* (totally new) scale.

It is important to emphasize that Table 3 represents only a theoretical mapping and does not constitute empirical validation of content validity. The cross-referencing demonstrates that each item has identifiable theoretical and diagnostic foundations, but whether these items function as intended in real populations remains an empirical question.

**Table 1:** Response Scale suggested by Claude AI for the first nine items of the Exercise Addiction Screening Scale-10 (EASS-10).

1	2	3	4	5	6
Never	Rarely	Sometimes	Often	Very often	Always

***Note:*** Response options range from 1 (*Never*) to 6 (*Always*) only for Items 1–9 of the EASS-10. Item 10 uses a binary Yes/No response format, and it is a critical criterion.

**Table 2:** Items, Symptoms, and Addiction Criteria of the Exercise Addiction Screening Scale-10 (EASS-10).

#	Item Wording	Symptom	Addiction Criterion
1	I exercise to escape my feelings of anxiety or tension.	*Withdrawal/Mood Modification*	Withdrawal
2	I continuously increase the intensity or duration of my exercise to achieve the desired effect.	*Tolerance*	Tolerance
3	I am unable to reduce the duration of my workouts even when I want to.	*Loss of Control*	Loss of Control
4	I typically exercise significantly more than I originally planned.	*Intention Effects*	Loss of Control
5	I continue to exercise even when I am injured or ill.	*Continuance Despite Harm*	Continuance
6	I prefer exercising over spending time with family or friends.	*Interpersonal Conflict/Salience*	Conflict
7	I think about my next workout even when I should be focusing on work or study.	*Cognitive Salience*	Salience
8	I have difficulty controlling my urge to exercise.	*Obsessive Passion/Compulsion*	Salience
9	I have the impression that exercise controls me rather than the other way around.	*Loss of Autonomous Control*	Loss of Control
10	In the past 3 months, has your exercise caused significant distress or problems in your work, relationships, physical health, or daily functioning? YES □ NO □	*Functional Impairment/Clinical criterion*	Clinical Significance

***Note:*** Items 1–9 are rated on a 6-point Likert scale (1 = Never, 6 = Always). Item 10 uses a Yes/No format and serves as a clinical gate; a “Yes” response is required for elevated item scores on statements 1–9 to indicate problematic exercise or risk of exercise addiction.

It should be acknowledged that the current item content has not been subjected to formal content validity assessment (e.g., expert panel review or Content Validity Index calculation). While the items were theoretically mapped onto established addiction frameworks and verified by the author as a domain expert, systematic content validation involving multiple independent experts is recommended as a priority step in future validation studies.

**Table 3:** EASS-10 Item-Level Cross-Reference: Theoretical Bases Across EDS-21, EAI (All Versions), Passion Scale, DSM-5, and ICD-11.

#	Item (Abbrev.)	EDS-21 Basis	EAI Basis	Passion Scale Basis	DSM-5 Basis	Primary Anchor
1	Escape anxiety/tension	Withdrawal/Affect regulation	Mood modification	OP, (emotional dependence)	Negative reinforcement use	Mood modification/OP
2	Increase intensity/duration	Tolerance	Tolerance	—	Tolerance criterion	Tolerance
3	Unable to reduce workouts	Lack of control	Relapse theme (implicit)	OP, (controlled internalization)	Unsuccessful efforts to cut down	Loss of control
4	More than planned	Intention effects	—	—	Larger/longer than intended	Impaired control
5	Continue despite injury	Continuance despite problems	EAI-3 injury item	OP, rigidity	Continued despite harm	Compulsive continuation
6	Prefer exercise over family	Reduction in other activities	Conflict	OP, conflict	Activities given up	Conflict/impairment
7	Preoccupation	Time/salience	Salience	OP, preoccupation	Craving/preoccupation	Salience
8	Difficulty controlling the urge	Lack of control	Implicit control theme	OP, loss of autonomy	Impaired control	Loss of control
9	Exercise controls me	Lack of control	Withdrawal-based urges	OP, internal pressure	Compulsive pattern	Compulsivity
10	Significant distress/impairment	—	—	—	Clinical distress/impairment required	Clinical gate

***Note:*** EAI = Exercise Addiction Inventory (Terry et al., 2004); EDS = Exercise Dependence Scale-21 (Hausenblas & Downs, 2002); OP = Obsessive Passion subscale of the Passion Scale (Vallerand et al., 2003); DSM-5 = Diagnostic and Statistical Manual of Mental Disorders, Fifth Edition (American Psychiatric Association, 2013); ICD-11 = International Classification of Diseases, Eleventh Revision (World Health Organization, 2019).

### 3.1. Scoring and Interpretation

Items 1–9 are scored on a 1–6 Likert scale (1 = Never, 6 = Always). Sum all nine items to obtain a total EASS-9 score (range: 9–54). Item 10 is binary (Yes/No) and functions as a clinical significance gate rather than contributing to the continuous score. The AI-generated analysis suggested that an elevated EASS-9 score in the absence of a “Yes” on Item 10 should be interpreted as an index of high-volume or passionate exercise rather than clinical addiction. Furthermore, the AI output initially suggested an incremental interpretation of the total scores: “*Scores between 9 and 26 indicate low risk, for which no clinical action is required. Scores of 27 to 36 reflect a moderate risk (provisional). If the impairment gate is endorsed (Yes), elevated concern is indicated, and brief counseling with reassessment in 4 weeks is recommended. Scores between 37 and 45 suggest high risk; if accompanied by a positive impairment response, probable addiction is indicated, and a clinical interview is recommended. Scores from 46 to 54 represent a very high risk; when combined with a positive impairment response, likely addiction is indicated, and referral for a structured clinical assessment is advised.*”

Additionally, the AI-generated output also suggested a two-stage screening protocol, which is more pragmatic for clinical screening. The following passage is quoted directly from the AI output: “Stage 1—Quantitative screen: Calculate the EASS-9 sum score. Scores ≥37 indicate high or very high risk and warrant further assessment. Stage 2—Clinical gate: If EASS-9 ≥ 37 AND Item 10 = Yes, the individual meets the threshold for probable exercise addiction and should be referred for a structured clinical interview or psychological assessment. If Item 10 = No, classify as “high-volume exerciser without demonstrated functional impairment” and monitor. This two-stage approach reduces the false-positive rate from approximately 15% (Likert-only) to less than 3% (note: these estimates were generated by the AI model based on theoretical assumptions and do not constitute empirically derived values; they should be treated as illustrative projections requiring empirical verification), aligning with the ≥90% screening certainty target specified in the development brief.”

However, when presented with the prompt “You propose these criteria, but from a clinical perspective, only the identification of those at risk of mental/psychiatric dysfunction is valuable for screening. In that case, what would be the reasonable score where you would recommend an interview follow-up?”, the AI model produced the following output (quote):

“This is an excellent and important clinical challenge to my proposed framework, and it deserves a direct answer. From a pure screening perspective, the clinically defensible single cutoff is ≥27, combined with Item 10 = Yes. Here is the reasoning:

The tiered system I proposed conflates screening with risk stratification—two different clinical purposes. For screening, whose sole goal is to identify who warrants a clinical interview, the tiered approach creates an unnecessary middle layer. The critical insight is this: once a person endorses Item 10 as Yes—meaning they are already experiencing functional impairment—the Likert severity score becomes secondary to the impairment itself. Impairment has been endorsed. That is your clinical signal, regardless of whether the score is 27 or 37.

A score of ≥27 represents a mean item response of ≥3 (Sometimes) across all nine addiction symptom dimensions. When this co-occurs with reported functional impairment, it constitutes a clinically meaningful presentation warranting a structured interview. Waiting for ≥37 risks two things: first, missing moderate cases where early intervention has the greatest impact; second, creating a false sense of security in the 27–36 range where impairment is already present.

The practical recommendation, therefore, is:

EASS-9 score ≥ 27 AND Item 10 = Yes → clinical interview indicated

Everything below that threshold, or where Item 10 = No, requires surveillance rather than clinical action. The low/moderate/high/very high gradations retain value for research and for communicating severity back to the individual, but they should not serve as the clinical decision gate. The gate is simpler: impairment present plus symptom threshold met equals refer.

This also aligns more honestly with how Lichtenstein et al. (2017) framed it—impairment is the necessary and sufficient clinical trigger, with symptom count providing supporting rather than determining evidence.”

First, the reference to Lichtenstein et al. is on the right track, but the more correct interpretation of their work would be: “*Lichtenstein et al. (2017) emphasize the importance of impairment (e.g., social and psychological harm) associated with compulsive exercise and highlight that quantitative symptom counts alone are insufficient for defining clinically significant dysfunction.*” So, the AI-generated reasoning is valid according to this paper, but it is somewhat imprecise in its interpretation. Lichtenstein et al.’s review underscores the need for future empirical research to clarify diagnostic criteria that meaningfully capture harmful consequences, rather than relying solely on symptom counts or questionnaire data.

Second, there is discordance in the suggested cutoff point between the tiered risk-stratification model (≥37 + impairment) and the later screening-for-referral model (≥27 + impairment), although this does not invalidate the logic. The inconsistency arises because the first framework yields severity classification with screening thresholds. Conversely, the latter separates them to yield a clinical criterion. In the first model, referral is recommended only at ≥37 with impairment, implying that moderate scores (27–36) with impairment require only brief counseling. However, the latter argument suggests that once functional impairment is identified (Item 10 = Yes), a mean symptom score ≥ 3 (total score ≥ 27) already indicates clinically meaningful dysfunction warranting follow-up interviews. From a psychometric and diagnostic perspective, the latter view is more consistent: screening aims to maximize sensitivity to identify cases that require further evaluation, whereas score bands for stratification are better suited to conveying severity or categorization in research. Thus, the apparent contradiction reflects a shift from a risk-stratification model with research purposes to a true screening approach, with impairment as a key clinical trigger and symptom load providing supporting evidence. Empirical testing is therefore necessary to validate or fine-tune these logic-based AI suggestions.

To reconcile this apparent inconsistency, the present paper adopts the screening-for-referral threshold of EASS-9 ≥ 27 combined with Item 10 = Yes as the primary clinical decision rule, as this approach maximizes sensitivity for identifying individuals who warrant further evaluation (Table 4). The tiered risk-stratification framework (low/moderate/high/very high) is retained for research and severity communication purposes only, not as the clinical screening gate. It is explicitly acknowledged that both thresholds are theoretically derived and require empirical calibration against structured clinical interview data before clinical deployment.

**Table 4:** Distinction between the Clinical Screening Rule and Descriptive Severity Classification for the EASS-10.

Function	Criterion	Interpretation	Recommended Use
Primary clinical screening decision	EASS-9 ≥ 27 and Item 10 = Yes	Clinical interview indicated	Primary clinical scoring rule
Low score	EASS-9 = 9–26	Low symptom endorsement; mean item response below “Sometimes.”	Research/severity description only
Moderate score	EASS-9 = 27–36	Moderate symptom endorsement; mean item response approximately “Sometimes” to “Often.”	Research/severity description only; clinical interview only if Item 10 = Yes
High score	EASS-9 = 37–45	High symptom endorsement; mean item response above “Often” to “Very often.”	Research/severity description only; clinical interview if Item 10 = Yes
Very high score	EASS-9 = 46–54	Very high symptom endorsement; mean item response above “Very often” to “Always.”	Research/severity description only; clinical interview if Item 10 = Yes

***Note:*** The score bands are provisional and descriptive only. They are derived from the arithmetic structure of the 1–6 response scale by converting total EASS-9 scores into approximate mean item endorsement levels. They are not empirically validated clinical thresholds and should be used only for research description or severity communication. The primary clinical screening rule remains EASS-9 ≥ 27 combined with Item 10 = Yes.

Thus, the EASS-9 score bands are not intended to override the primary clinical decision rule; they are provided only to describe symptom severity in research or feedback contexts. From the perspective of classical screening theory, the choice of a lower threshold (≥27) over a higher one (≥37) reflects a deliberate prioritization of sensitivity over specificity. In screening contexts, the cost of missing a true case (false negative) typically outweighs the cost of a false alarm (false positive), because individuals flagged by screening are subsequently evaluated through clinical interview, which corrects for false positives. Conversely, false negatives receive no follow-up and remain undetected. This sensitivity-prioritizing logic, well established in diagnostic screening methodology (Streiner et al., 2024), supports adopting the lower threshold for clinical screening, with the understanding that specificity will be formally evaluated once empirical data are available.

### 3.2. Theoretical Justification for EASS-10

The AI-recommended EASS-10 is based on verified theoretical and empirical research in EA. The scale incorporates the six-component framework of behavioral addiction proposed by Griffiths (2005), which defines addictive behavior through symptoms like salience, mood modification, tolerance, withdrawal, conflict, and relapse. This framework, despite criticism (Fournier et al., 2023), is widely used in exercise addiction research and provides a structured approach to measuring maladaptive exercise habits. It is worth noting that although the EASS-10 includes the components model, it is not based solely on it. Additionally, the distinction between HP and OP helps differentiate high engagement from compulsive behavior. Studies show that OP is consistently linked to EA, while HP may not be consistently associated with it (Kovacsik et al., 2018; Szabo et al., 2022). Concerns about inflated prevalence rates in cross-sectional questionnaire studies have been raised by Szabo (2024), who observed large discrepancies between clinically significant cases and survey results. The EASS-10, which assesses functional impairment, can reduce the likelihood of false-positive interpretations of EA risk. At the same time, the EASS-10 addresses Lichtenstein et al.’s (2017) emphasis on the importance of functional impairment in untangling EA risk from healthy exercise. Overall, these insights support a screening approach grounded in theory and evidence, with a focus on clinical relevance rather than just counting symptoms, and support the logic underlying the EASS-10.

A specific note is warranted regarding Item 8 (“I have difficulty controlling my urge to exercise”), which captures obsessive passion (OP) at the symptom level. While the extant literature has predominantly positioned OP as a predictor of EA rather than a component (Szabo & Kovacsik, 2019; Kovacsik et al., 2018), the present instrument intentionally repositions OP-related content as a symptom indicator. The theoretical rationale is threefold: (1) the phenomenological experience of impaired control over exercise urges, regardless of its etiological origin in obsessive passion, constitutes a clinically observable symptom that maps directly onto DSM-5 impaired control criteria; (2) given that OP could account for up to 50% of the variance in EA (Kovacsik et al., 2018), excluding OP-related content from a screening tool would omit a strong signal of problematic exercise; and (3) within a screening context, OP-related content can be included not as evidence that obsessive passion is a defining component of exercise addiction, but as a clinically relevant risk indicator that may help identify individuals warranting further assessment, consistent with the use of prediction-oriented indicators in screening instruments (Streiner et al., 2024). Nevertheless, future validation studies should empirically examine whether Item 8 functions as a distinct OP indicator or loads equivalently with other addiction-symptom items, and whether repositioning OP as a moderator rather than a component improves predictive validity.

### 3.3. Testing the EASS-10 on AI-Generated Simulated Data

To illustrate whether the prespecified unidimensional simulation model could generate internally coherent ordinal response patterns, a Monte Carlo simulation was performed, generating N = 500 cases using a unidimensional Graded Response Model (GRM; Samejima, 1969) to model ordinal Likert-type responses. The simulated data were analyzed with confirmatory factor analysis in R (*lavaan* v. 06-21; Rosseel, 2012) employing the WLSMV estimator, which is recommended for ordinal indicators. A single-factor model was specified *a priori*, with all nine Likert-type items (Items 1–9) loading onto a general EA factor, consistent with the theoretical framework. Model fit was assessed based on established criteria (CFI and TLI ≥ 0.95, RMSEA ≤ 0.06, SRMR ≤ 0.08; Hu & Bentler, 1999).

The following simulation parameters were used: item discrimination parameters (a) ranged from 0.5 to 1.2, and item difficulty (threshold) parameters (b) were set to span −1.5 to 1.5 on the latent trait continuum, reflecting expected variation in symptom endorsement across a general exercising population. These values were selected based on typical parameter ranges observed in comparable behavioral addiction screening instruments. Latent trait scores were drawn from a standard normal distribution N(0, 1). The sample size of N = 500 was chosen because it exceeds the minimum recommended for stable WLSMV estimation with ordinal indicators (Bandalos, 2014) and provides adequate statistical power to detect model misfit in CFA with 9 indicators.

Using Jamovi software (2.6.44), the unidimensional model demonstrated excellent global fit: *χ*^2^(27) = 33.00, *p* = 0.199; CFI = 0.981; TLI = 0.974; RMSEA = 0.021 (90% CI [0.000, 0.043]); SRMR = 0.030. All standardized factor loadings were statistically significant (*p* < 0.001), ranging from 0.296 to 0.573, indicating low-to-moderate associations between the indicators and the latent construct. Residual correlations were uniformly small (ranging from −0.073 to 0.065), suggesting no substantial local dependence or systematic model misfit. Because these analyses were conducted on *simulated* covariance structures derived from theoretically specified parameters, the results support the structural coherence and plausibility of a unidimensional representation but *do not constitute empirical validation*.

An important methodological caveat is warranted: because the simulated data were generated from a unidimensional GRM and then analyzed using a unidimensional CFA, the excellent fit indices are, to a degree, tautologically expected. The simulation, therefore, serves as a computational plausibility check—confirming that the specified item parameters produce internally coherent data—rather than independent structural validation. Additionally, five standardized factor loadings fell below the conventional adequacy threshold of 0.40 commonly recommended for clinical screening instruments (Hair et al., 2019). In the simulated CFA, these were Item 4 (“I typically exercise significantly more than I originally planned”; λ = 0.384), Item 5 (“I continue to exercise even when I am injured or ill”; λ = 0.366), Item 6 (“I prefer exercising over spending time with family or friends”; λ = 0.362), Item 7 (“I think about my next workout even when I should be focusing on work or study”; λ = 0.340), and Item 8 (“I have difficulty controlling my urge to exercise”; λ = 0.296). While these lower values may partly reflect deliberately heterogeneous symptom coverage across distinct addiction dimensions, empirical validation is needed to confirm whether these items demonstrate sufficient discrimination in real samples. These items should therefore be treated as priority candidates for empirical review, including possible wording revision, theoretical retention if conceptually necessary, or removal if poor discrimination is confirmed in real validation samples.

To state this point with maximal clarity: the excellent global fit indices (CFI = 0.981, TLI = 0.974, RMSEA = 0.021) reported in this section are an unavoidable arithmetic consequence of analyzing data generated under the same unidimensional model that was subsequently tested. These values carry no inferential weight regarding the EASS-10’s performance in human populations and should not be interpreted as evidence of structural validity. Their sole function is to confirm that the simulation procedure was correctly implemented and that the specified item parameters are internally coherent.

Taken together, these findings suggest that the AI-generated EASS-10, synthesized from the EDS, EAI, and PS, appears theoretically consistent, pending empirical confirmation. The satisfactory model fit supports the conceptual integration of impaired control, tolerance, salience, conflict, continuance despite harm, and obsessive internalization within a single latent factor consistent with behavioral addiction frameworks. However, empirical testing with large, heterogeneous exercise samples remains essential. Future validation should include confirmatory factor analysis, measurement invariance testing, reliability estimation (e.g., omega coefficients), and criterion validation against structured clinical interviews. If theoretical assumptions hold, one might expect adequate internal consistency (α/ω ≥ 0.80), replication of the unidimensional or closely related hierarchical structure, meaningful associations with obsessive passion and stress, and potentially improved specificity relative to single-instrument approaches due to the embedded impairment gate.

### 3.4. Discriminant Validity Considerations

An important limitation of the current proof-of-concept is the absence of a discriminant validity assessment. The functional impairment gate (Item 10) could be endorsed by individuals whose distress stems from conditions other than exercise addiction, including compulsive exercise secondary to eating disorders (Lichtenstein et al., 2014), excessive exercise driven by body dysmorphia, or generalized psychological distress unrelated to exercise behavior. Each of these conditions shares the impairment criterion with EA and could inflate false-positive screening rates if the EASS-10 is administered without additional clinical context. Future validation studies should therefore include concurrent measures of eating disorder symptomatology (e.g., the Eating Disorder Examination Questionnaire), compulsive exercise (e.g., the Compulsive Exercise Test; Taranis et al., 2011), and general psychological distress (e.g., the Depression Anxiety Stress Scales; Lovibond & Lovibond, 1995) to establish that the EASS-10 demonstrates adequate discriminant validity. Additionally, the impairment gate should be examined for its specificity to exercise-related functional impairment rather than general psychosocial impairment.

### 3.5. Limitations and Future Directions

The EASS-10 is a theoretically derived instrument that requires empirical validation before clinical deployment. Specific limitations include: (a) the item selection is based on construct mapping rather than item response theory (IRT) analysis of an existing combined-item pool; (b) the proposed cutoff scores are derived from parent-scale literature and require calibration in a sample with gold-standard structured interview data; (c) the binary gate (Item 10) may reduce sensitivity in populations with poor insight into their own impairment (common in exercise addiction given the ego-syntonic nature of the behavior—individuals may not recognize exercise as causing their relational or occupational difficulties).

Additional limitations warrant acknowledgment: (d) the AI model’s training data are opaque, introducing potential biases in item generation that cannot be fully audited; although the author verified all items against established frameworks, systematic biases in AI-generated content remain a concern in AI-assisted research (Bender et al., 2021); (e) the instrument has not undergone formal content validation by an independent expert panel, which is a standard step in scale development (DeVellis & Thorpe, 2021); and (f) integrating items from three distinct source scales (EDS, EAI, PS) carries a risk of construct contamination, whereby the resulting composite may inadvertently conflate related but distinct constructs. Future validation should include confirmatory analyses specifically designed to evaluate whether the EASS-10 measures a unitary construct or an inadvertent composite of exercise dependence, exercise addiction, and obsessive passion.

Finally, it must be reiterated that the present study constitutes a proof-of-concept demonstration of AI-assisted instrument development. The complete absence of empirical participant data is acknowledged as the primary limitation. All psychometric indicators reported (fit indices, factor loadings) are products of simulated data and carry no inferential weight regarding the instrument’s real-world performance. The contribution of this work lies in the methodological process (demonstrating AI-assisted theoretical synthesis) and its theoretical product (a structurally coherent screening tool), both of which are offered as a foundation for the empirical validation studies that must follow.

Future validation studies should: apply the EASS-10 in exercising samples with concurrent structured clinical interview (SCID-based or exercise addiction-specific protocol); conduct IRT analysis to confirm item difficulty and discrimination parameters; evaluate measurement invariance across sex, exercise type, and culture; and establish sensitivity, specificity, positive and negative predictive values against clinical diagnosis.

## 4. Conclusions

This paper examines the capacity of AI-assisted synthesis (using Claude AI) to systematically combine established theoretical frameworks and validated tools into a cohesive screening method for EA. By integrating the EDS-21, EAI, and the OP subscale of the PS within a single, clinically grounded structure that includes a functional impairment threshold, the AI-generated EASS-10 aims to address key methodological issues in the field, such as the overestimation of EA risk prevalence due to the lack of tools with diagnostic criteria and insufficient consideration of obsessive passion.

The simulated psychometric evaluation is consistent with the internal structural plausibility of a unidimensional model; however, these findings remain *theoretical* until confirmed through empirical samples. Therefore, the main task for researchers in this field is rigorous real-world validation: testing the EASS-10 against structured clinical interviews, assessing reliability and measurement invariance across cultures and exercise types, establishing optimal screening cutoffs, and directly comparing its sensitivity and specificity with existing tools. Only through such empirical validation can the field determine whether AI-assisted scale synthesis can meaningfully enhance the assessment of EA risk for prevention purposes beyond traditional instrument development methods.

It should be noted that the structural plausibility demonstrated by the Monte Carlo simulation is logically, not empirically, established. The simulation confirmed that the specified item parameters are internally coherent under the assumed model, but this does not constitute evidence that the EASS-10 will replicate this structure in real-world samples. Prioritized next steps for validation include: (a) criterion validity testing against structured clinical interviews in clinical and exercise populations; (b) item response theory analysis to confirm item difficulty and discrimination parameters; (c) measurement invariance evaluation across sex, exercise type, and culture; and (d) direct comparison of sensitivity and specificity with existing EA instruments.

## Data Availability

The data are available upon reasonable request from the author.

## References

[B1-behavsci-16-00817] American Psychiatric Association (2013). Diagnostic and statistical manual of mental disorders.

[B2-behavsci-16-00817] Bandalos D. L. (2014). Relative performance of categorical diagonally weighted least squares and robust maximum likelihood estimation. Structural Equation Modeling.

[B3-behavsci-16-00817] Bender E. M., Gebru T., McMillan-Major A., Shmitchell S. (2021). On the dangers of stochastic parrots: Can language models be too big?. Proceedings of the 2021 ACM conference on fairness, accountability, and transparency.

[B4-behavsci-16-00817] Chhabra B., Árok P., Szabo A. (2024). A cross-cultural examination of passion and risk of exercise addiction in Hungarian and Indian exercisers. International Journal of Sport and Exercise Psychology.

[B5-behavsci-16-00817] Chhabra B., Demetrovics Z., Griffiths M. D., Szabo A. (2026). Exercise addiction: A review and evaluation of current research and theory. Journal of Behavioral Addictions.

[B6-behavsci-16-00817] Chyung S. Y., Roberts K., Swanson I., Hankinson A. (2017). Evidence-based survey design: The use of a midpoint on the Likert scale. Performance Improvement.

[B7-behavsci-16-00817] de la Vega R., Almendros L. J., Barquín R. R., Boros S., Demetrovics Z., Szabo A. (2022). Exercise addiction during the COVID-19 pandemic: An international study confirming the need for considering passion and perfectionism. International Journal of Mental Health and Addiction.

[B8-behavsci-16-00817] de la Vega R., Parastatidou I. S., Ruíz-Barquín R., Szabo A. (2016). Exercise addiction in athletes and leisure exercisers: The moderating role of passion. Journal of Behavioral Addictions.

[B9-behavsci-16-00817] DeVellis R. F., Thorpe C. T. (2021). Scale development: Theory and applications.

[B10-behavsci-16-00817] Downs D. S., Hausenblas H. A., Nigg C. R. (2004). Factorial validity and psychometric examination of the Exercise Dependence Scale–Revised. Measurement in Physical Education and Exercise Science.

[B11-behavsci-16-00817] Fournier L., Schimmenti A., Musetti A., Boursier V., Flayelle M., Cataldo I., Starcevic V., Billieux J. (2023). Deconstructing the components model of addiction: An illustration through “addictive” use of social media. Addictive Behaviors.

[B12-behavsci-16-00817] Granziol U., Griffiths M. D., Zou L., Yang P., Herschel H. K., Junker A., Akimoto T., Stoll O., Alpay M., Aydın Z., Zandonai T., Di Lodovico L., Lichtenstein M. B., Trott M., Portman R. M., Schipfer M., Cook B., Cerea S., Egorov A. Y., Szabo A. (2023). The expanded exercise addiction inventory (EAI-3): Towards Reliable and international screening of exercise-related dysfunction. International Journal of Mental Health and Addiction.

[B13-behavsci-16-00817] Griffiths M. D. (2005). A “components” model of addiction within a biopsychosocial framework. Journal of Substance Use.

[B14-behavsci-16-00817] Hair J. F., Black W. C., Babin B. J., Anderson R. E. (2019). Multivariate data analysis.

[B15-behavsci-16-00817] Hausenblas H. A., Downs D. S. (2002). Exercise dependence: A systematic review. Psychology of Sport and Exercise.

[B16-behavsci-16-00817] Hu L. T., Bentler P. M. (1999). Cutoff criteria for fit indexes in covariance structure analysis: Conventional criteria versus new alternatives. Structural Equation Modeling: A Multidisciplinary Journal.

[B17-behavsci-16-00817] Kovacsik R., Soós I., de la Vega R., Ruíz-Barquín R., Szabo A. (2018). Passion and exercise addiction: Healthier profiles in team than in individual sports. International Journal of Sport and Exercise Psychology.

[B18-behavsci-16-00817] Lichtenstein M. B., Christiansen E., Elklit A., Bilenberg N., Støving R. K. (2014). Exercise addiction: A study of eating disorder symptoms, quality of life, personality traits and attachment styles. Psychiatry Research.

[B19-behavsci-16-00817] Lichtenstein M. B., Hinze C. J., Emborg B., Thomsen F., Hemmingsen S. D. (2017). Compulsive exercise: Links, risks and challenges faced. Psychology Research and Behavior Management.

[B20-behavsci-16-00817] Lovibond S. H., Lovibond P. F. (1995). Manual for the depression anxiety stress scales.

[B21-behavsci-16-00817] Marsh H. W., Vallerand R. J., Lafrenière M.-A. K., Parker P., Morin A. J. S., Carbonneau N., Jowett S., Bureau J. S., Fernet C., Guay F., Salah Abduljabbar A., Paquet Y. (2013). Passion: Does one scale fit all? Construct validity of two-factor passion scale and psychometric invariance over different activities and languages. Psychological Assessment.

[B22-behavsci-16-00817] Mónok K., Berczik K., Urbán R., Szabó A., Griffiths M. D., Farkas J., Magi A., Eisinger A., Kurimay T., Kökönyei G., Kun B., Paksi B., Demetrovics Z. (2012). Psychometric properties and concurrent validity of two exercise addiction measures: A population wide study. Psychology of Sport and Exercise.

[B23-behavsci-16-00817] Rosseel Y. (2012). lavaan: An R package for structural equation modeling. Journal of Statistical Software.

[B24-behavsci-16-00817] Samejima F. (1969). Estimation of latent ability using a response pattern of graded scores. Psychometrika Monograph Supplement.

[B25-behavsci-16-00817] Streiner D. L., Norman G. R., Cairney J. (2024). Health measurement scales: A practical guide to their development and use.

[B26-behavsci-16-00817] Sun J., Lu T., Shao X., Han Y., Xia Y., Zheng Y., Wang Y., Li X., Ravindran A., Fan L., Fang Y., Zhang X., Ravindran N., Wang Y., Liu X., Lu L. (2025). Practical AI application in psychiatry: Historical review and future directions. Molecular Psychiatry.

[B27-behavsci-16-00817] Szabo A. (2024). Chasing a phantom dysfunction: A position paper on current methods in exercise addiction research. International Journal of Mental Health and Addiction.

[B28-behavsci-16-00817] Szabo A., de la Vega R., Kovácsik R., Jiménez Almendros L., Ruiz-Barquín R., Demetrovics Z., Boros S., Köteles F. (2022). Dimensions of passion and their relationship to the risk of exercise addiction: Cultural and gender differences. Addictive Behaviors Reports.

[B29-behavsci-16-00817] Szabo A., Griffiths M. D., de la Vega R., Mervo B., Demetrovics Z. (2015). Methodological and conceptual limitations in exercise addiction research. Yale Journal of Biology and Medicine.

[B30-behavsci-16-00817] Szabo A., Kovacsik R. (2019). When passion appears, exercise addiction disappears. Swiss Journal of Psychology.

[B31-behavsci-16-00817] Taranis L., Touyz S., Meyer C. (2011). Disordered eating and exercise: Development and preliminary validation of the Compulsive Exercise Test (CET). European Eating Disorders Review.

[B32-behavsci-16-00817] Terry A., Szabo A., Griffiths M. D. (2004). The exercise addiction inventory: A new brief screening tool. Addiction Research & Theory.

[B33-behavsci-16-00817] Vallerand R. J., Blanchard C., Mageau G. A., Koestner R., Ratelle C., Léonard M., Gagné M., Marsolais J. (2003). Les passions de l’âme: On obsessive and harmonious passion. Journal of Personality and Social Psychology.

[B34-behavsci-16-00817] World Health Organization (2019). International classification of diseases, eleventh revision (ICD-11).

